# Tumour Seeding After Transbronchial Cryobiopsy in a Patient With Newly Diagnosed Lung Cancer

**DOI:** 10.1002/rcr2.70193

**Published:** 2025-05-07

**Authors:** Sammy Onyancha, Isabelle Dettmer, Njuxhersa Maloku, Gernot Rohde

**Affiliations:** ^1^ Department of Pulmonology St. Elisabethen Krankenhaus Frankfurt Germany; ^2^ Medical Clinic I, Department of Respiratory Medicine Goethe University Frankfurt, University Hospital Frankfurt Germany

**Keywords:** adenosquamous cell carcinoma, bronchoscopy, lung cancer, transbronchial cryobiopsy, tumour seeding

## Abstract

Tumour seeding following biopsy is a rare but clinically significant complication in lung cancer diagnostics, particularly in aggressive malignancies. A 66‐year‐old male with a significant smoking history and known chronic obstructive pulmonary disease presented with chronic cough and weight loss. Imaging revealed a central airway mass with mediastinal lymphadenopathy. Bronchoscopic evaluation and biopsy confirmed the diagnosis of metastatic non‐small cell lung cancer with high PD‐L1 expression. Shortly after the initial procedure, the patient developed airway obstruction due to a rapidly growing endobronchial lesion at the prior biopsy site, confirmed histologically as tumour seeding. Multiple bronchoscopic interventions were required to restore airway patency. Despite prompt escalation to combined chemo‐immunotherapy and repeated multimodal airway recanalisation, the disease demonstrated aggressive local recurrence. This case highlights the potential for endobronchial tumour seeding following diagnostic interventions in lung cancer, underscoring the importance of vigilant follow‐up and prompt management in cases with rapid disease progression.

## Graphical abstract

Tumour seeding following biopsy is a rare but clinically significant complication in lung cancer diagnostics, particularly in aggressive malignancies. This case highlights the potential for endobronchial tumour seeding following diagnostic interventions in lung cancer, underscoring the importance of vigilant follow‐up and prompt management in cases with rapid disease progression.

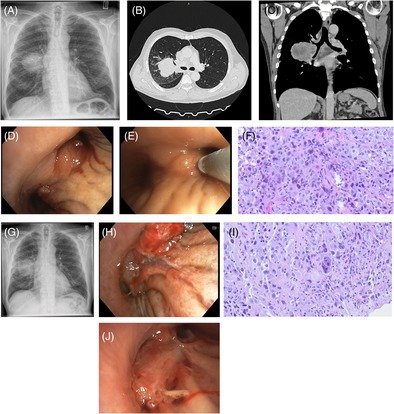

## 1 Introduction

Bronchoscopic biopsies are a key diagnostic tool for lung cancer. While generally safe, tumour cell implantation at the procedural site, known as tumour seeding, is a rare but significant complication [1, 2]. This phenomenon has been reported in association with transthoracic needle aspiration, pleural drainage, and surgical incisions, but it remains less commonly described in bronchoscopic procedures.

## 2 Case Report

A 66‐year‐old male, an active smoker with a 50‐pack‐year history, presented with a persistent cough and unintentional weight loss of 8 kg over 2 months. He had a history of chronic obstructive pulmonary disease but no known malignancy.

**FIGURE 1:**
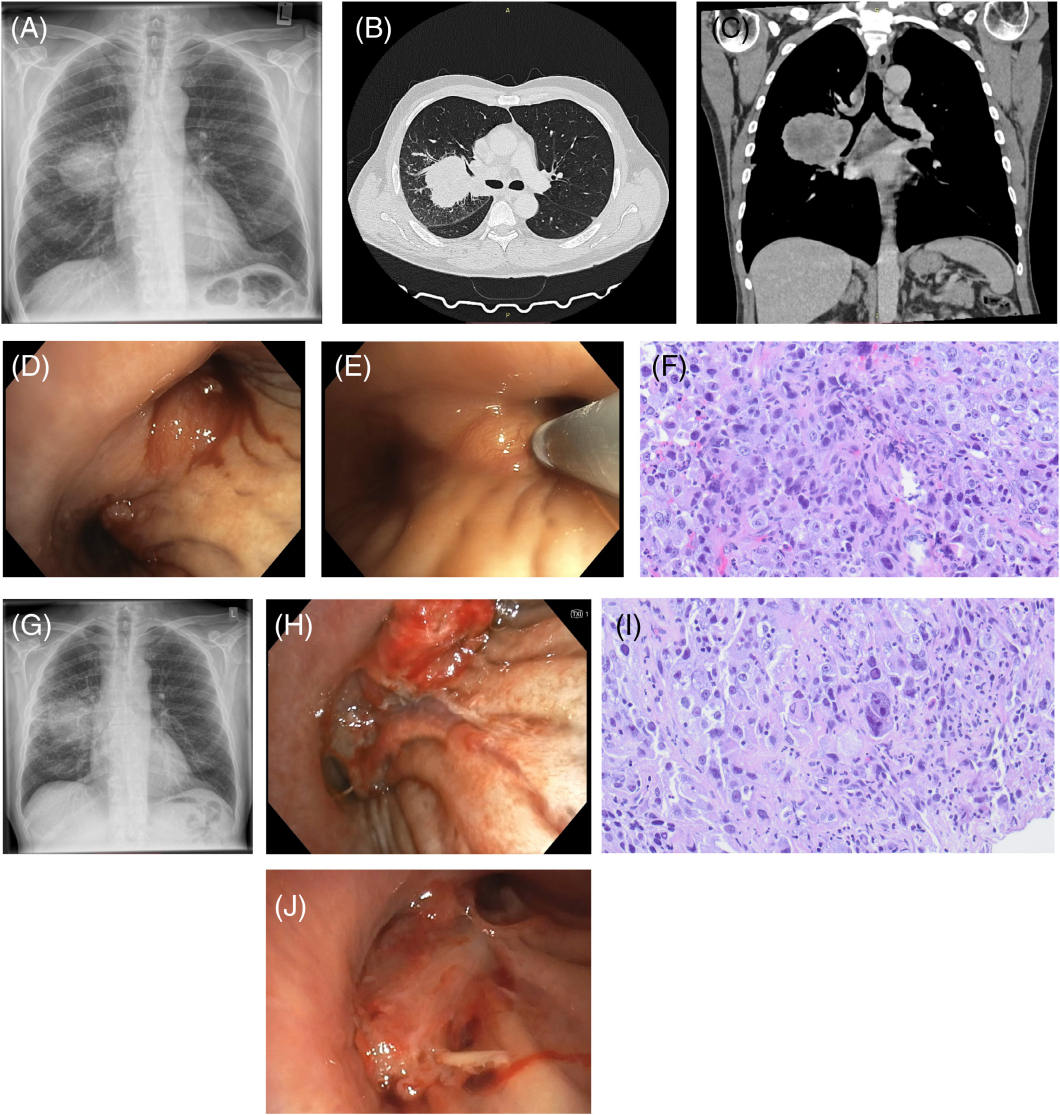
(A) Initial chest x‐ray showing central mass in the right lung. (B, C) CT‐scan showing suspicion of lung cancer in the right lung. (D) Bronchoscopy revealing exophytic lesion with changes in bronchial mucosa. (E) Cryobiopsy of infiltrated bronchial mucosa. (F) HPE of mucosal cryobiopsy showing the presence of adenocarcinoma. (G) Chest x‐ray 10 days after biopsy showing right‐sided pneumonia and slight growth of tumour mass. (H) Repeat bronchoscopy revealing endobronchial growth causing airway obstruction of the right lung. (I) HPE of cryoextraction of the endobronchial growth showing adenocarcinoma. (J) Repeat bronchoscopy showing recurrence of endobronchial tumour growth.

Initial imaging with chest x‐ray (Figure 1A) and follow‐up CT scan (Figure 1B,C) revealed a central airway mass in the right upper lobe accompanied by mediastinal lymphadenopathy. Diagnostic flexible bronchoscopy revealed an exophytic endobronchial lesion at the right upper lobe carina with clear evidence of mucosal infiltration (Figure 1D). The airway remained patent, allowing inspection down to the subsegmental bronchi. Endobronchial ultrasound‐guided transbronchial needle aspiration was performed for mediastinal staging, and transbronchial cryobiopsy was obtained from the endobronchial lesion (Figure 1E). No procedural complications were observed, and the postinterventional chest x‐ray showed no signs of pneumothorax.

Histopathological evaluation confirmed the presence of non‐small cell lung cancer, specifically adenocarcinoma, with immunohistochemistry positive for thyroid transcription factor 1 (Figure 1F).

PD‐L1 expression was high at 80% and tumour staging revealed cerebral and intra‐abdominal metastases. The patient was discharged and scheduled to begin immunotherapy monotherapy, pending molecular profiling results.

However, 1 week post‐bronchoscopy, he developed rapidly worsening dyspnoea and was readmitted. Repeat chest x‐ray showed pneumonia of the right lung and subtle signs of tumour growth (Figure 1G).

Repeat bronchoscopy revealed a new, irregularly growing lesion at the site of the previous biopsy, extending into the right main stem bronchus, occluding the middle and lower lobe segments (Figure 1H). Airway recanalisation was done using a biopsy snare and cryoextraction, with the patient's condition rapidly improving after reestablishment of airway patency.

Histopathological analysis of the newly extracted tissue revealed tumour cells with an identical immunohistochemical profile to the primary lesion (Figure 1I), consistent with endobronchial tumour seeding following biopsy.

Given the aggressive disease course, systemic treatment was escalated to combined chemo‐immunotherapy, which was initiated the day following airway recanalization. However, despite the prompt initiation of therapy, the patient's condition deteriorated within 2 weeks. Due to clinical signs of airway obstruction and worsening hypoxemia, a rigid bronchoscopy was done, which revealed a recurrence of the tumour growth into the right main stem bronchus (Figure 1J). Repeat airway recanalization was performed using cryotherapy, and argon plasma coagulation (APC) was applied to the affected mucosa to reduce the risk of further tumour regrowth (Video 1). Due to the rapid tumour recurrence, the multidisciplinary board decided to proceed with local radiotherapy.

**VIDEO 1:** Video summary of the various endoscopic interventions in the case. Video content can be viewed at https://onlinelibrary.wiley.com/doi/10.1002/rcr2.70193

## 3 Discussion

This case highlights a rare but significant complication of bronchoscopic biopsy procedures: endobronchial tumour seeding. While flexible bronchoscopy with cryobiopsy is a well‐established technique for obtaining high‐quality diagnostic tissue, particularly in peripheral lesions, the potential for iatrogenic dissemination of malignant cells through mechanical manipulation remains a concern, especially in tumours with high proliferative potential.

Endobronchial seeding remains an exceedingly rare phenomenon, with only sporadic case reports in the literature pertaining to infectious diseases [3, 4, 5]. In this case, the timeline and characteristics of the secondary lesion (directly at the site of prior biopsy and sharing identical histopathological features with the primary tumour) support the conclusion that biopsy‐related tumour implantation was the underlying mechanism. Several factors may have contributed to this outcome, including the exophytic nature of the primary lesion, mucosal disruption during biopsy, and the use of cryoextraction, which can leave denuded surfaces that may be conducive to tumour implantation.

Moreover, the patient's immunocompromised status due to malignancy and the presence of concomitant pneumonia likely contributed to the accelerated local tumour growth and clinical deterioration.

Notably, the recurrence of airway obstruction within days of initial recanalisation and the need for repeat bronchoscopic interventions underscores the aggressive nature of this case. The use of cryotherapy and APC proved effective in re‐establishing airway patency and mitigating immediate respiratory compromise, although these were ultimately temporising measures in the context of rapidly progressive disease.

While bronchoscopic biopsy remains essential for diagnosis and staging, clinicians should maintain a high index of suspicion for post‐procedural tumour progression, especially when new endobronchial lesions emerge at or near instrumentation sites. Timely recognition and intervention are critical to managing airway compromise.

Although preventive strategies are not well established, minimising excessive biopsy trauma, avoiding unnecessary biopsies in highly friable tumours, and early detection through surveillance bronchoscopy in high‐risk cases may help reduce the risk of tumour seeding.

Future studies may help identify risk factors as well as establish preventive measures and optimal management strategies. Awareness of this phenomenon is essential for early diagnosis and appropriate therapeutic modifications.

## Author Contributions

All the authors contributed to the manuscript. The first draft of the manuscript was written by Sammy Onyancha, and all the authors commented on previous versions of the manuscript. All the authors have read and approved the final manuscript.

## Ethics Statement

The authors declare that written informed consent was obtained for the publication of this manuscript and accompanying images using the consent form provided by the journal.

## Conflicts of Interest

The authors declare no conflicts of interest.

## Data Availability

The data that support the findings of this study are available from the corresponding author upon reasonable request.
